# Weed Seed Decay in No-Till Field and Planted Riparian Buffer Zone

**DOI:** 10.3390/plants9030293

**Published:** 2020-03-01

**Authors:** Nebojša Nikolić, Andrea Squartini, Giuseppe Concheri, Piergiorgio Stevanato, Giuseppe Zanin, Roberta Masin

**Affiliations:** Department of Agronomy, Food, Natural Resources, Animals and Environment, University of Padova, 35020 Legnaro (PD), Italy; squart@unipd.it (A.S.); giuseppe.concheri@unipd.it (G.C.); stevanato@unipd.it (P.S.); giuseppe.zanin@unipd.it (G.Z.); roberta.masin@unipd.it (R.M.)

**Keywords:** weed seed bank, no-till management, soil microbiological activity, seed degradation

## Abstract

Field management practices can alter the physical and chemical properties of the soil, also causing changes to the seed bank. Alterations can also occur to the soil microbial community, which in turn can increase or diminish the process of weed seed decay. In this research, the issue of seed degradation was studied in an undisturbed and a no-till soil, trying not only to uncover where seeds are more degraded, but also to investigate the microbial activities that could be involved in this process. Six different weed species, commonly found in northern Italy, were used: *Abutilon theopharsti*, *Alopecurus myosuroides*, *Amaranthus retroflexus*, *Digitaria sanguinalis*, *Portulaca oleracea* and *Sorghum halepense*. Seed decay was tested in two different sites, a no-till field and the adjacent buffer zone. Soil microbial activity was also measured using the Fertimetro, an approach based on the degradation of cotton and silk threads buried in the soil for one week. Degradation of the buried seeds was higher in the no-till field soil than in the buffer strip for all the studied species as was the microbial cellulolytic activity. Even though the buffer strip soil is an undisturbed habitat and resulted as having higher organic matter, the no-till soil conditions appeared more unfavourable to seed viability. Our findings suggest that no-till management can improve weed seed suppression in the soil. Moreover, cellulolytic microorganisms play an important role in seedbank longevity, so cellulolytic activity surveys could be used as an early monitoring bioindicator for weed seed suppression in soil.

## 1. Introduction

Weeds are problematic for agricultural production on a global level. They are naturally strong competitors, very often having the upper hand over crops in competing for resources. They can also interfere with harvesting, produce nocuous substances and serve as hosts for different harmful organisms [1].

Once in the soil, seeds of weed species tend to create stocks, better known as seed banks. Seeds in these seed banks represent the potential for infestation. Studies concerning biology, phenology and longevity of weed seeds in the soil are fundamental for determining the most efficient control strategies. Persistence and longevity of seeds in the soil are one of the most studied weed species characteristics, since they can be determining factors for colonization capacity. By maintaining viability while waiting for favourable conditions for germination, seeds with higher longevity in the soil could be a source of infestation year after year. Seed longevity in the soil seed bank is a species-dependent characteristic, but it can also largely depend on soil conditions and management [2,3]. Seeds also interact with soil microorganisms that can promote or inhibit seed germination. Although sometimes underestimated, the influence of microorganisms can be quite significant, given that they can produce plant-suppressive compounds, decrease plant available nutrients, colonize the seeds and cause plant diseases or act directly on seeds in the case of organic matter decomposers [4]. Seed longevity in the soil can, therefore, be strongly influenced by the number and variety of soil microorganisms. This is particularly true in soil with a tendency for nutrient accumulation, which can lead to soil microorganism proliferation. Other factors that can influence the soil microbial community are: vegetation cover and its rhizosphere, water content, temperature and climate. Some factors such as soil particle sizes, aggregate characteristics, depth of soil, and nutritional content can have a high impact on soil microbial population distribution and community structure. Discontinuities of these factors can create different microbial communities even in small spaces and relatively close to each other [5,6].

In agriculture, different cultivation systems, field-management practices and infestation control methods can change or alter physical and chemical properties of the soil, also causing changes to the seed bank and the soil microbial community, which in turn can increase or diminish the process of seed decay [2,3]. In conventional agriculture diverse tillage operations are used to maintain high crop productivity and keep weed seeds present in the soil under control by burying them in deeper layers and thus preventing their germination, or bringing them to the surface and leaving them vulnerable to predation or causing premature emergence leading to seedling death. At the same time, these practices lead to changes in soil structure and distribution of nutrients, so changes of soil microbial communities are also possible. Although the main purpose of tillage practices is improvement of the soil physical conditions to grow crops, they can leave the soil vulnerable to erosion, desiccation and less organic matter that can lead to soil infertility [7,8,9,10]. Using the practices of conservation agriculture (CA), farmers are fighting against these negative effects on the soil caused by tillage in conventional agriculture [11,12].

Three fundamental aspects of CA are minimal soil disturbance, permanent soil cover with crop residues and live mulches, and crop rotation. Due to the benefits that CA provides, an increasing number of farmers are adopting these practices and in 2015/2016 the land under CA reached 180 million ha worldwide [13]. After the adoption of CA, farmers indicate weed management as one of the most difficult operations [9,14,15,16]. Indeed, due to minimum or no tillage operations, weed seeds remain in the soil surface layer developing a larger seed bank likely to emerge more quickly than in conventional systems, where the seeds are buried deeper [17]. In particular mechanical weeding is never applicable when no-till practices are adopted, so weed control relies only on herbicides and agronomic practices. This suggests that in no-till farming integrated weed management is the best option to deal with the problem of weeds. Therefore in no-till more than in other tillage systems, understanding of the ecological and biological aspects of weeds can help to achieve an efficient weed management [18]. However, there is still not enough research that tackles the issue of seed longevity and decay in soils under CA [9,14,15,19].

Many studies in the past have found that agricultural soils with a high level of biological activity can be weed-suppressive [20,21,22]. Undisturbed and less disturbed soils have higher biological activity than disturbed ones [23]. This is probably due to the differences in the content of soil nutrients, micro and macro elements, organic matter, pH value and conductibility, characteristics that can also influence the presence, variety and distribution of soil microorganisms as well as their activity. Thus a greater difference in seed decay is expected between disturbed and undisturbed soils, where undisturbed soils are reported to be more weed-suppressive [24].

In this research we approached the issue of seed degradation in undisturbed or minimally disturbed soils used for different purposes, trying not only to uncover where seeds are more degraded, but also to understand the reasons behind this process. Seed decay of six different weed species was tested in two adjacent sites, a field under no-tillage and a buffer zone. The six species selected are common weeds in the fields of the Northern Italy, belong to different families, and have different seed size and seed coat thickness, which are the features that could influence the degradation process. The two experimental sites were selected for their close proximity and different usage, in order to compare the seed-decay process between a completely undisturbed, pristine habitat—the buffer strip, and a minimally disturbed field, but used for agricultural purposes, the no-tillage field. Soil microbial activity was also measured during the burial period to evaluate the role of microorganisms in seed degradation. This paper, therefore, aims to provide a multi-faceted insight into the dynamics of seed degradation between differently managed sites with diverse soil disturbance and the microorganism activity that can influence these dynamics in the soil. A dual methodological approach was undertaken that would allow the results obtained from two independent standpoints to be crossed: the seed fate on one side and the enzymatic microbial activity recorded on different substrate baits (cotton and silk). The final purpose is to improve knowledge on the seed bank fate in the field under conservation agriculture to optimize weed management in sustainable cropping systems.

## 2. Results

### 2.1. Fate of the Buried Seeds

**Figure 1:**
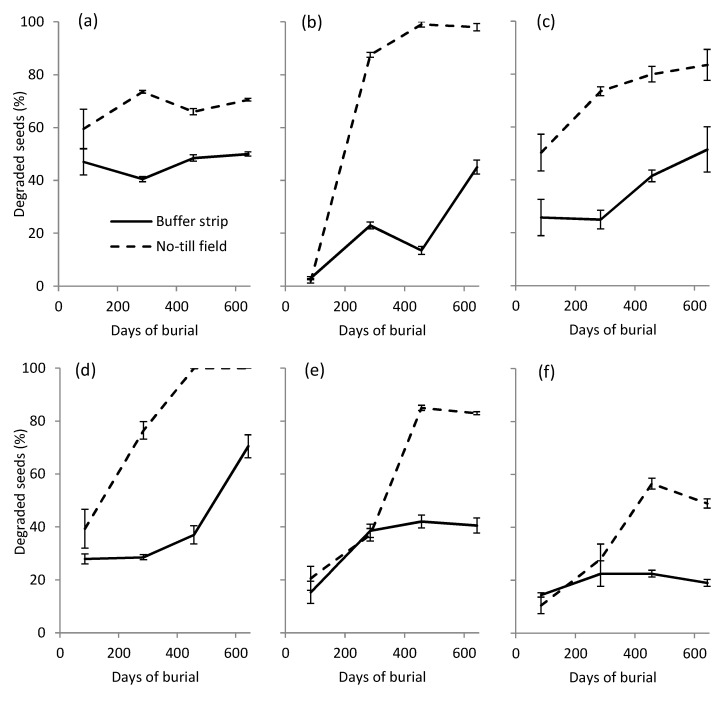
Percentage of degraded seeds after different days of burial in the soil of the no-till (NT) field and vegetated buffer strip (BS). (**a**) *A. theophrasti*; (**b**) *A. myosuroides*; (**c**) *A. retroflexus*; (**d**) *D. sanguinalis*; (**e**) *P. oleracea*; (**f**) *S. halepense*.

Significant main effects and interactions were detected on both seed degradation and viability percentage (*p* < 0.01). Degradation of the buried seeds was higher in the no-till (NT) field soil for all the studied species (Figure 1). The most degraded species in the NT field were *Digitaria sanguinalis* and *Alopecurus myosuroides*, while the least degraded were *Abutilon theophrasti* and *Sorghum halepense*. As in the NT soil, the most degraded species in the buffer strip (BS) was *D. sanguinalis*, with 70% of degraded seeds after 643 days of burial, while the least degraded was *S. halepense*, with only 19% of degraded seeds at the end of the experiment. *A. theophrasti*, a species with a thick and hard seed coat, had degraded fast at the first exhumation in both sites, but in the next exhumations the percentage of seed degradation remained stable at around 70% in the field and 50% in the BS.

**Figure 2:**
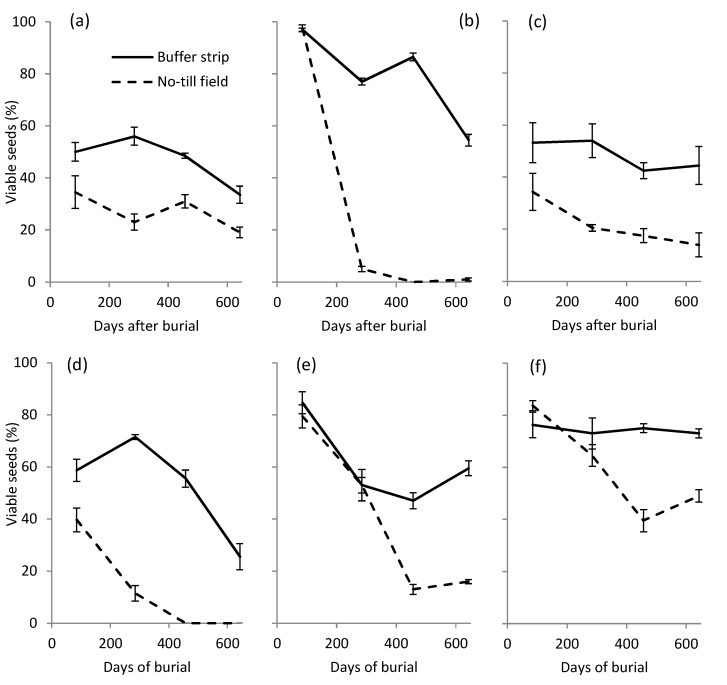
Percentage of viable seeds after different days of burial in the soil of the NT field and vegetated BS. (**a**) *A. theophrasti*; (**b**) *A. myosuroides*; (**c**) *A. retroflexus*; (**d**) *D. sanguinalis*; (**e**) *P. oleracea*; (**f**) *S. halepense*.

The seeds of all six species buried in the BS soil were still viable after 643 days of burial, while the species buried in the NT field soil had totally (*A. myosuroides* and *D. sanguinalis*) lost their viability or had a residual survival of 10–20% of buried seeds, only *S. halepense* maintained a viability of about 50% of buried seeds (Figure 2).

### 2.2. Soil Microbial Activity

**Figure 3:**
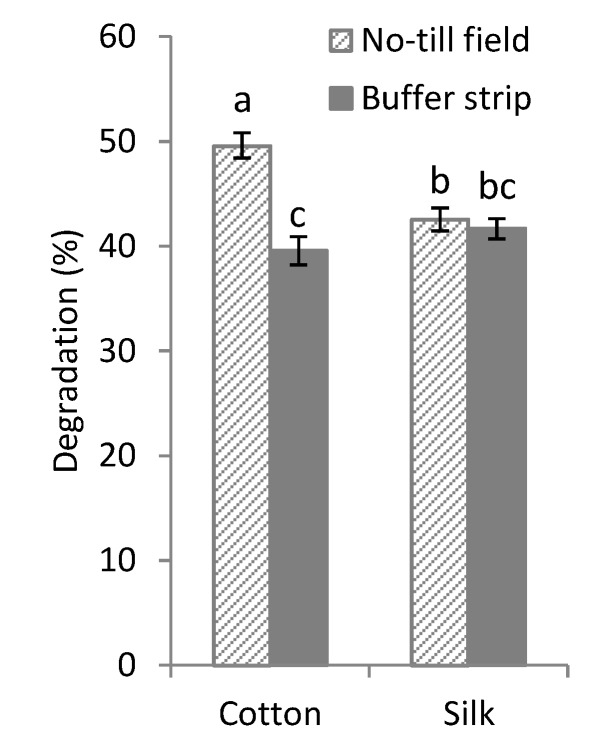
Degradation percentage of mean values of both treated and non-treated cotton and silk threads of Fertimetro observed in the NT field and vegetated BS. Pairs of data sharing the same letter are not significantly different (*p* > 0.05). Bars report the standard error.

Cotton threads of Fertimetro were more degraded in the NT field than in the BS, indicating a greater activity of cellulolytic microorganisms in the NT field with respect to the BS. In the BS, degradation of both silk and cotton threads was not significantly different, indicating similar activity of proteolytic and cellulolytic microorganisms, unlike in the NT field where cellulolytic activity was higher than proteolytic (Figure 3). Data shown are the mean values of both control (non-treated) and treated cotton and silk threads of the Fertimetro.

**Figure 4:**
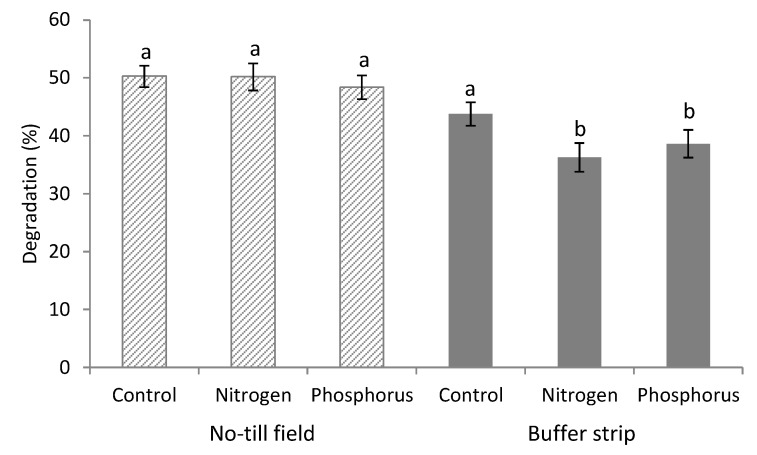
Degradation percentage of Fertimetro cotton threads observed in the NT field and vegetated BS. Control: untreated thread; Nitrogen: thread pre-treated with N solution; Phosphorus: thread pre-treated with P solution. Pairs of data sharing the same letter are not significantly different (*p* > 0.05). Bars report the standard error.

In the NT field there were no differences in degradation of control and pre-treated threads, indicating no major deficiencies for N or P. In the BS control, threads were more degraded than those treated with N and P solutions, indicating an abundance of these nutrients in the soil of this habitat, indeed the microbes found the nutrients in the soil for their metabolism and they are less able to respond to further N and P addition, whose levels become excessive on the treated threads (Figure 4).

**Figure 5:**
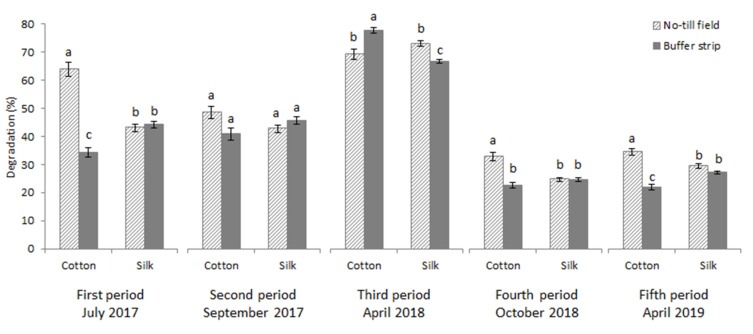
Degradation percentage of mean values of both treated and non-treated cotton and silk threads of Fertimetro buried in different periods in the soil of the NT field and the vegetated BS. Pairs of data sharing the same letter are not significantly different (*p* > 0.05). Bars report the standard error.

**Figure 6:**
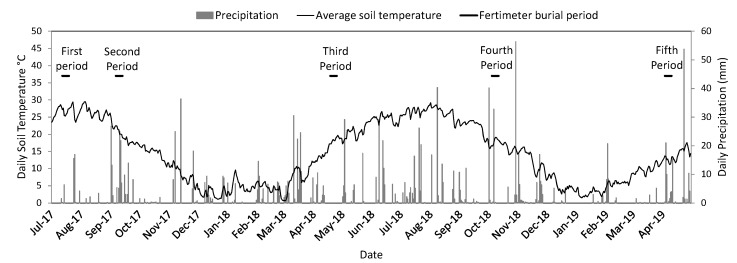
Meteorological data during the trial period with the Fertimetro burial periods indicated. Soil temperature was measured at the depth of 10 cm.

The observation of the percentages of Fertimetro degradation in the five burial periods, using the mean values of both control (non-treated) and treated cotton and silk threads of the Fertimetro, (Figure 5) reveals the relationship with the meteorological data (Figure 6). The period with higher degradation is the third (in April 2018), characterized by high temperatures and low precipitation, low soil humidity corresponded to a higher soil microbial activity, supposedly due to a lower value of water-filled pore space which allows higher rates of aerobic respiration. In this period, the silk was degraded more in the NT field than in the BS and the cotton was degraded more in the BS than in the NT field. All the other periods were characterized by rainfall, the microbial activity was lower than in the drier period for both fibres, with higher degradation of cotton in the NT field, whereas the silk was degraded similarly in the NT field and in the BS.

## 3. Discussion

In this work our aim was to assess the effects of the soil management variable on weed seeds’ longevity by combining a plant-based and a microbe-based type of analysis to provide evidence of the possible correlated interplay of these two living members of the system and the differences accountable by plant species and by preferred degraded substrate from the microbial celluloytic and proteolytic guilds.

After less than two years of burial, marked differences appeared in seed viability between the NT field and BS and among weed species. Only seeds of *D. sanguinalis* and *A. myosuroides* in the NT soil totally lost their viability, all the other species were still viable with a percentage varying from 20% to 80%. Although a comparison with the literature results is difficult due to different seed burial conditions and experimental protocols, it is interesting to observe that all six species studied in our experiment are often classified as long-lived (seeds surviving ≥ 3 years) [25,26]. In accordance with this classification, many studies found seeds of these same species still viable in the soil after 2 years [27,28,29,30] in different habitats, even the seeds of *D. sanguinalis* [27,29,31] and *A. myosuroides* [30,32]. For these two species, therefore, the seed longevity detected in NT soil appears particularly short, indicating that the conditions they experienced under this soil management are particularly unfavourable to their viability. Physical properties of seeds seemed to be a key characteristic for determining the level of degradation, the species with a thicker seed coat were the least degraded (*A. theophrasti*, *S. halepense*), while those with thinner seed coats (*D. sanguinalis*, *A. myosuroides*) were more degraded. These results are confirmed by the findings of [29] and [33], who found a positive relationship between seed coat thickness and seed viability in the soil. Thus, seed coat is an effective barrier preserving the seed against external aggression by abiotic and biotic factors, the most important of which is microbial attack [33]. In our study, soil microbiological activity of the NT field showed high activity of cellulolytic microorganisms that feed on the seed coat, mostly made of polyphenolic polymers such as lignin and cellulose [34,35,36,37]. The lower cellulolytic activity in the BS soil would justify the reduced degradation of seeds in this habitat.

Considering only the microbial activities, it is particularly interesting to observe that the NT field that has not been tilled in the past four years has already developed an apparently recovered status up to attaining the levels of a non-agriculturally perturbed habitat such as the woody BS. In our study the highest microbial activity was detected in the NT field not in the BS, although the latter habitat was undisturbed by farming and showed higher organic matter (Table 1). These findings seem to be in contrast to those of [20] who defined weed suppressive soils as those with a higher level of organic matter, where generally higher numbers of potentially weed-suppressive microorganisms were recovered. To explain this one can consider that the different types of habitat may be related with either saprophytic activity or temperature, that is known to interact with substrate chemistry on cellulosic activity. As alternative, and less likely hypothesis, one could assume that the soil of the woody BS would probably be particularly rich in cellulosic matter and the microorganisms could, therefore, be less prone to colonize the seeds. While in the NT field, even though crop residues remain on the soil surface, cellulosic matter in the soil is scarcer resulting in greater attention for seeds by microorganisms that can therefore influence the process of seed decay more strongly than in the BS. If our hypothesis can be confirmed, we could suggest the use of cellulolytic activity surveys as a valuable early monitoring bioindicator for weed seed suppression in soil.

The fact that cotton degradation was regularly higher in the field in comparison to the buffer strip with the exception of the third measurement (April 2018), which followed a drier period, is in line with a more intense activity of cellulolytic microbes under the very efficient aerobic respiration metabolism. Since undisturbed and continuously vegetated buffer strips are known to feature a superior water infiltration capacity in comparison to cropped fields, the latter are those where aerated conditions (and apparent drought) would more often be the case. But under a real drought challenge, as the third period shows, it appears that the buffer strip environment, offering a better soil structure and therefore retaining water reserves for life also when pore spaces would be mostly filled with air, the ensuing enzymatic activity on cellulose proves superior to that attained in the NT field.

The NT field, in comparison to its adjacent uncropped buffer strip, proved to be superior in terms of abating weed seeds viability for all tested species over a nearly two-year period, pushing most of them down to a residual survival rate below 20%.

It is important to underline that in our experiment, seeds were placed at a depth of 12 cm to avoid germination, but most weed seeds in a NT soil are in the surface layer. Therefore, for a deeper understanding of weed seedbank dynamics in a NT field, it is fundamental to also investigate seed fate in the 0–5 cm layer where the germination process occurs, preventing lethal infection from pathogens. Thus, in the surface layer, microbial damage of the seed coat could lead not only to microbial penetration and degradation of seed content but also make the coat permeable to water, accelerating the germination process. Moreover, in the surface layer, the role of seed dormancy becomes crucial to determine seed survival in the soil, as hypothesized by [38] who defined the dormancy-defence syndromes, according to which seeds rely on distinct set of pathogen defenses depending on the types of dormancy (physical, physiological and quiescence).

## 4. Materials and Methods

### 4.1. Experimental Site

The experiment was conducted from 2017 to 2019 at the experimental farm of the University of Padova in northeast Italy (45°12’ N, 11°58’ E, altitude 6 m a.s.l.). The climate of the area is sub-humid with a mean annual temperature of 15.6 °C with a cold winter and hot summer, and mean annual rainfall of about 850 mm. The experiment started in summer 2017 in two adjacent areas: a field under no-till management since 2014 and the buffer zone on the field boundary. The parent rock consists of alluvial deposits from extremely calcareous clays and silts. The three soils are classified as Fluvic Cambisols (Calcaric, Hypereutric, Oxyaquic, Orthosiltic) following the criteria of the FAO-UNESCO system [39,40] and as Oxyaquic Eutrudept fine-silty, mixed, mesic according to the Soil Taxonomy [40,41]. They are typical soils of the recent low plain of the Veneto region with a moderate deep, olive brown colour and no gravel. Their texture is clay loam with alkaline reaction, and a high carbonate content is present through the entire profile depth. Information about the soil properties can be found in Table 1. Soil physicochemical properties were analysed as previously described [42]. All results are expressed on an oven-dry basis. At the beginning of the experiment, the soil under NT was covered with wheat residues. Soybean was sown in July and harvested in October 2017. In November 2017, horseradish was sown as a cover crop then eliminated with a herbicide treatment in April. In May 2018 the field was sown with maize, and harvested in mid-September. Wheat was grown from November 2018 till the end of the experiment. The adjacent buffer strip (BS) was 6 m wide with two rows of trees (*Platanus hybrida* Brot.) and bushes (*Viburnum opulus* L.). In the space between the two rows there was no herbaceous cover due to the lack of sunlight caused by the tree crowns, while a mulch cover derived from fallen leaves was present.

Meteorological data during the experiments were monitored from the Regional Agency for Environmental Protection (ARPA) meteorological station located in the farm.

### 4.2. Seed Burial and Classification

Mature seeds of six weed species, *Abutilon theophrasti*, *Alopecurus myosuroides*, *Amaranthus retroflexus*, *Digitaria sanguinalis*, *Sorghum halepense* and *Portulaca oleracea*, were collected in summer 2016 from natural populations growing on the farm. The inflorescences of mature plants were hand-harvested, cleaned and mature seeds selected. The seeds were dry-stored at room temperature until burial. For every species 32 groups of 50 seeds were inserted in small bags made from very dense steel mesh nets in order to keep in the seeds, but at the same time, allow a normal flow of air and water in and out. All the bags with seeds were buried randomly on 12 July 2017, 16 bags for each species in the field and 16 in the buffer strip, in holes of 70 × 70 cm dug in the ground at a depth of 12 cm to avoid germination.

The holes were refilled with the soil in order to minimize the disturbance and recreate the original covering.

The trials were set to last for 21 months with 4 exhumations, each one with 4 replications for each species, the first after 3 months (in October 2017) and then every 6 months until April 2019.

After every exhumation, the seeds were tested, first they were classified as intact if they remained firm after squeezing with a pair of tweezers [43]. Those that failed the test were marked as degraded, those that passed (intact seeds) were subjected to a germination test, placed in Petri dishes with 2 mL of distilled water and put in an incubator at 25/15 °C and 12/12 h dark/light photoperiod. The germination process was monitored every 2–3 days. After a few weeks the non-germinated seeds were stored at 4 °C for four weeks and then again placed in the incubator with optimal temperature for germination. After twice in the incubator, the tetrazolium test was performed on the non-germinated seeds to control their viability [43]. Ultimately, the seeds were classified as degraded, germinated, dormant (vital under tetrazolium test) and non-viable. Percentages of buried seeds degraded and viable (germinated + dormant) were calculated.

Factorial analysis of variance (ANOVA) was performed to analyse the effect of site, exhumation time and species and their interactions on percentage of degraded and viable seeds. Data were arcsine of square root transformed to achieve homogeneity of variances. Values were back-transformed to be presented in the Results section with their original measure unit.

### 4.3. Soil Microbial Activity

Microbial activity of the soils was tested using Fertimetro, as described by [42,44,45]. The method consists of evaluating the degradation of cotton and silk threads buried in the soil for one week. The different fibers provide the assessment of cellulolytic (cotton) and proteolytic (silk) microbial activities. Some threads are pre-treated with N or P solutions, the comparison between the degradation of the control non-treated threads and treated ones indicates the availability or deficiency of the nutrients (N and P) in soil.

Fertimetro threads were buried in the soil for 7 days in both sites before every exhumation of the seeds. After burial, the threads were exhumed, air dried and their resistance to breakage tested using a dynamometer and compared to an unburied thread. The resistance percentage was then converted into the degradation percentage as a complement to 100%.

A Kruskal–Wallis test was performed to analyse the effect of site and treatment on degradation percentage of silk and cotton threads. Significant differences among means were identified using Dunn’s multiple comparison test.

## Figures and Tables

**Table 1:** Soil physicochemical properties of the studied sites (C: organic carbon; Cc: carbonate content; CEC: cation-exchange capacity).

Site	Texture (%) Clay Silt Sand			pH	C (%)	N (%)	C/N	Cc (%)	CEC (cmoL^(+)^kg)
BS	29	31	40	8.37	1.81	0.18	10.2	28.3	28.5
NT	31	31	38	8.45	1.36	0.13	10.3	28.6	26.3
